# Quality Improvement Interventions across a Network of Pediatric Hematology–Oncology Clinics

**DOI:** 10.1097/pq9.0000000000000149

**Published:** 2019-03-11

**Authors:** Jennifer Morgan, Jeffrey Deyo, Jennifer Cox, Francisca Fasipe, Ashraf Mohamed, Carolyn Russo

**Affiliations:** From the * Affiliate Program Office, St. Jude Children’s Research Hospital, Memphis, Tenn.; † St. Jude Baton Rouge Affiliate Clinic, Our Lady of the Lake Regional Medical Center, Baton Rouge, La.; ‡ St. Jude Affiliate Clinic at Huntsville Hospital for Women and Children, Huntsville, Ala.; § St. Jude Affiliate Clinic at Mercy Children’s Hospital—Springfield, Springfield, Mo.; ¶ St. Jude Affiliate Clinic at The Children’s Hospital at Saint Francis, Tulsa, Okla.

## Abstract

**Introduction:**

Achieving improvement in quality among different institutions is challenging. Immunocompromised children with febrile neutropenia are at high risk of severe infection. Pediatric hematology–oncology patients frequently experience central line–associated bloodstream infections (CLABSIs) associated with implanted catheters. A network of 8 pediatric hematology–oncology clinics affiliated with St. Jude Children’s Research Hospital launched 2 initiatives designed to reduce the incidence of infections and improve infection treatment.

**Methods:**

We reviewed the timing of antibiotic administration for immunocompromised patients with a fever before and after a quality improvement intervention tailored to each affiliate clinic. We also reviewed the frequency of CLABSIs before and after implementing a central line care bundle for implanted catheters in ambulatory patients.

**Results:**

Across the affiliate clinic network, the timing of antibiotic administration improved from the preintervention period (23% of patients received antibiotics within 60 min of registration) to 53% and 73%, in successive postintervention periods. Implementing a central line bundle for implanted catheters was associated with increased compliance and a trend toward increased time between CLABSIs.

**Conclusion:**

We describe an approach to quality improvement utilizing a system of monitoring with annual clinical audits, development of joint quality improvement initiatives, ongoing education, and focused training of staff for effecting change that improves patient healthcare across multiple institutions.

## Abstract 2

Supplemental Digital Content is available in the text.

## INTRODUCTION

The affiliate program at St. Jude Children’s Research Hospital (St. Jude) was established to minimize referrals when affiliate clinics could provide equivalent care. The affiliate program allows more children to gain access to novel treatment strategies afforded by a large research hospital. Currently, 8 clinics are affiliated with St. Jude. The clinics are located throughout the Southeastern and the Midwestern United States, and together contribute 35% of the patients enrolled in St. Jude–led clinical trials. All 8 clinics serve mainly rural and suburban communities with broad patient demographics. The combined number of new oncology patients in all 8 affiliate clinics is approximately 350 patients per year.

The optimal training of staff requires periodic travel for direct face-to-face learning opportunities. For the St. Jude network, we provide face-to-face learning annually to the staff from different sites with a similar background in process improvement. Each affiliate site has a dedicated nurse educator and a clinic medical director to champion quality efforts. The local champions receive support and guidance from the affiliate program nurse director who functions as the team leader. Each clinic is expected to complete 2 quality improvement projects each year.

Although partnerships with affiliate clinics provide many opportunities to improve patient care, maintaining consistent and uniform levels of quality across a network can be challenging.^1,2^ To address these challenges, the affiliate program at St. Jude conducts annual clinical audits of each affiliate site. The auditors review clinical documentation, clinical observation of patient care, and outcome assessments, in addition to clinical trial compliance (see **Table S1, Supplementary Digital Content 1**, which displays the clinical observations during annual audits of affiliate clinics, http://links.lww.com/PQ9/A74). When deficiencies in an audit are identified, quality improvement interventions are initiated across the network, because issues identified at one affiliate site may exist at other sites even in the absence of a noted deficiency. This report describes 2 case studies to address deficiencies from recent clinical audits. Both projects were implemented in the context of a Plan-Do-Study-Act (PDSA) framework.

## CASE STUDY A: TIME TO ANTIBIOTICS IN FEBRILE IMMUNOCOMPROMISED PATIENTS

### Background

An audit reported that a patient with sepsis experienced a delay in administration of antibiotics resulting in prolonged hospitalization and a delay in chemotherapy. Immunocompromised children with febrile neutropenia are at high risk of severe infection.^3^ Prompt administration of broad-spectrum antibiotics for immunocompromised children with fever and neutropenia is a nationally recognized benchmark.^4,5^ The goal of this project was to ensure that 90% of febrile, immunocompromised patients receive antibiotics within 1 hour of their registration time in the healthcare setting, a “time to antibiotic” intervention. We performed a retrospective analysis to assess antibiotic administration timing in immunocompromised patients with fever. Of the 8 sites, 6 participated in this project.

### Methods

#### Program-wide Interventions.

Each affiliate program initiated a PDSA process to improve timely antibiotic administration for children with febrile neutropenia. The intervention started with a literature review and review of clinical best practices that were carried out by the affiliate nursing director together with a lead physician. This information was shared and discussed with all affiliate sites via webinars and shared slide decks. Follow-up education with each site occurred during monthly teleconferences.

### Site-specific Interventions

Each site-specific team was comprised the nurse educator, clinic medical director, clinic nurses, clinic physicians, and a pediatric pharmacist. During the period of literature and best practices review, baseline data were extracted retrospectively from medical records for a start time from patient registration to an end time of initiation of antibiotics. These data were submitted in a survey format to the nursing director for the affiliate program. From site medical records, each site’s nurse educator extracted data for 10 patients who were treated for febrile neutropenia at their affiliate clinic.

Each affiliate team created 2 process maps that outlined how febrile, immunocompromised patients entered the individual healthcare system during the clinic hours and after hours. Patients entered the healthcare system in a variety of ways, through the clinic directly, through emergency departments, or by direct admission to an inpatient unit. There was a separate process map for each location. The process maps included the following details:

1. How the patient contacted the clinic?
2. Where the patient arrived and how they were assessed?
3. How the nurse and clinician were notified?
4. How laboratory exams and antibiotics were ordered and if order sets were available?
5. How supplies for intravenous access and laboratory tests were obtained?
6. How pharmacy and laboratory staff were notified?
7. How antibiotics were prepared and delivered to the patient’s location?
8. How antibiotics were administered to the patient, and how the patient was reassessed?

Each affiliate team reviewed the process maps with the affiliate nursing director. Time measurements of each step helped each affiliate team identified the steps in the process causing a delay in care. Each site developed a plan to improve its timing of antibiotic treatment and tailored each plan to the site-specific process maps. The affiliate clinics without electronic order entry developed order sets with standardized dosing according to the most recently recorded patient weight. The affiliate clinics with electronic order entry developed a system to activate orders when notified of fever before the patient’s arrival. Every site implemented education through weekly team meetings of physicians, nurses, and pharmacists regarding the importance of prompt assessment and administration of antibiotics in immunocompromised patients with fever. During each performance cycle, we reinforced the plans during monthly conference calls among all the sites; the monthly calls included a review of ongoing performance.

As described below, time to antibiotics after an initial PDSA cycle consisting of education and the development of order sets improved, but not to the goal of 90%. We recognized that education needed to be extended beyond the clinic staff to the emergency department and after-hours staff. Awareness and education were helpful for this group of community physicians who were less familiar with fever in immunocompromised children and comprised a second PDSA cycle.

#### Study of Interventions

The preintervention period of this project spanned from April 1, 2012 to May 31, 2013. This period encompassed the retrospective chart review of baseline data, the literature review, the development of the process maps, and the design of each clinic’s intervention to eliminate delays in antibiotic administration. By July 1, 2013, all sites started their intervention according to their specific process mapping. From July 1, 2013 to December 1, 2013 was postintervention period 1 following the first intervention of initial education and the implementation of the order sets. After a review of the first postintervention data, we expanded education to include the emergency department and inpatient staff, and then we collected the second cycle of data. Postintervention period 2 was from January 1, 2014 to March 31, 2014.

#### Measures

We measured time to antibiotic administration as the difference between the time stamp of patient registration and the initiation of antibiotic infusion. This measure was recorded by clinic nurses and reported to the affiliate nursing director on a monthly basis.

#### Statistical Analysis

We compared differences between the 3 intervention periods for the time with the antibiotic using one-way ANOVA and pairwise *t* tests adjusted for multiple testing.

### Results

**Fig. 1.**
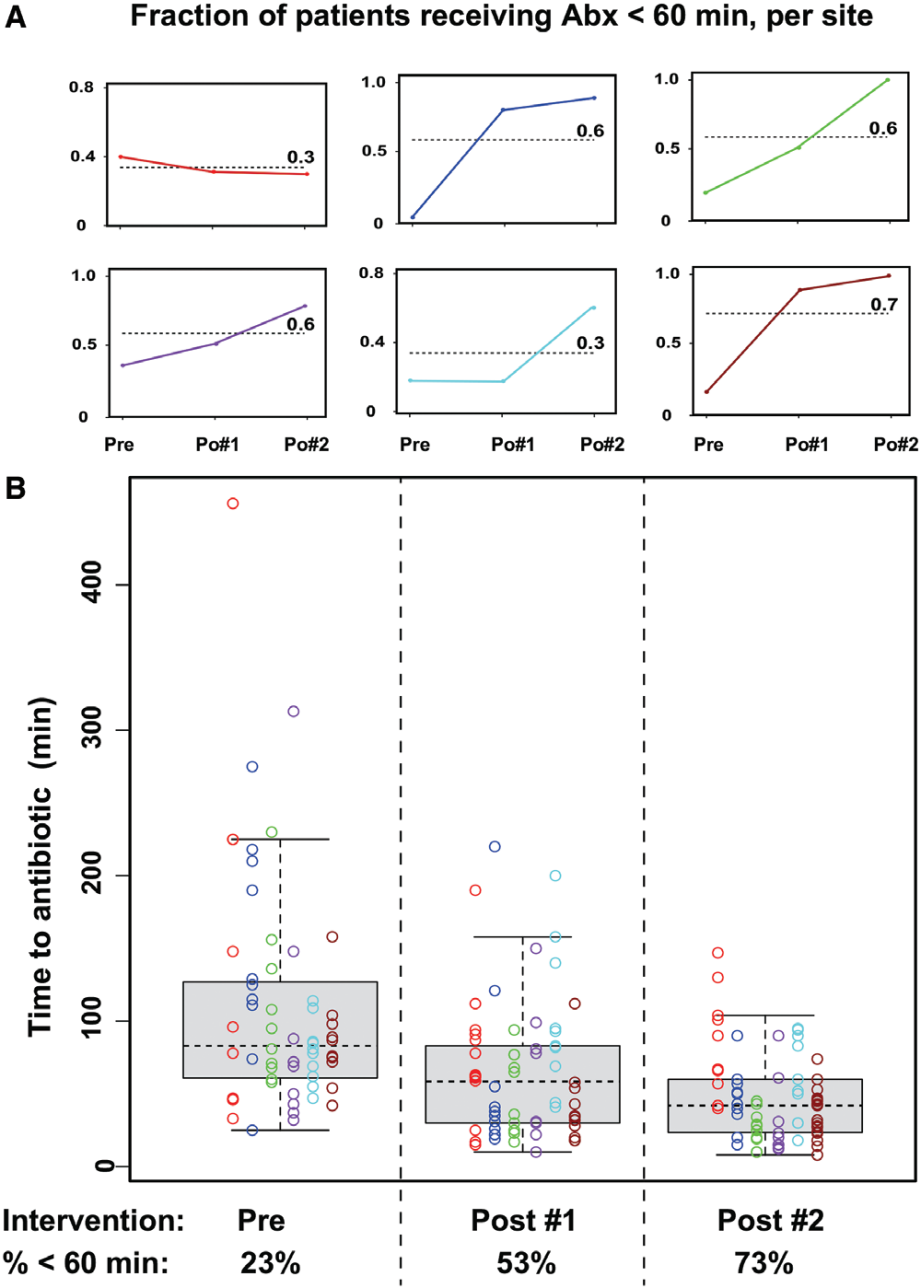
Effect of site-specific interventions on antibiotic administration. A, Fraction of patients receiving antibiotics within 60 minutes per site in Pre vs Post 1 vs Post 2. B, Time between patient registration and the initiation of antibiotic infusion is shown for 193 events, according to when the patient was seen (Preintervention, or in 2 successive postintervention periods, as described in the text), and the affiliate site (color). Data across all sites are summarized in a box and whisker plot for each period, and also as the percentage of patients receiving antibiotics in less than 60 minutes. Differences between successive periods are highly significant (Pre vs Post 1, *P* = 0.000008; Post 1 vs Post 2, *P* = 0.009, pairwise *t* test adjusted for multiple testing).

Between April 2012 and May 2013 (the preintervention period) of 60 immunocompromised patients with fever treated with antibiotics, 14 received antibiotics within 60 minutes of patient registration (23%). Between July 2013 and December 2013, the proportion increased to 53% (35 of 66), still short of our goal of 90%. During a review of the episodes, we identified the need to educate after-hours and emergency department staff. Therefore, we provided a second intervention of education to these groups, and between January 2014 and March 2014, the proportion of patients receiving antibiotics within 60 minutes of patient registration increased to 72% (49 of 67). Run charts for the individual sites who participated in this project are shown in Figure 1A. Individual time to antibiotic values are shown for every patient across all 3 periods in Figure 1B, and the aggregate difference between successive periods is highly significant (Pre versus Post 1, *P* = 0.000008; Post 1 versus Post 2, *P* = 0.009).

### Discussion

These results demonstrate a clear effect of implementing a quality improvement intervention for the time to administration of antibiotics in immunocompromised patients. An important aspect of our results is that the approach to improve time to antibiotic administration varied among the affiliate sites. Upon reviewing the differences between the process maps, we realized that in some sites, children were evaluated initially in the emergency department, but in other sites, the initial evaluation occurred in the inpatient unit. Moreover, some clinics had electronic order entry and some (at the time of this project) relied on paper charting. Importantly, the process map differences lead to site-specific approaches for improving quality of care. For example, at sites in which children are initially evaluated in the emergency department, improvement involved maintaining readily available standard doses of antibiotics for emergency department staff to administer. By contrast, at sites in which children are initially evaluated in inpatient units, quality improvement was implemented by developing a prepared order set with a preregistration system. These observations underscore the utility of process maps in improving patient care across a clinical network.

## CASE STUDY B REDUCTION OF CENTRAL LINE–ASSOCIATED BLOODSTREAM INFECTIONS

### Background

A second audit reported that the number of central line–associated bloodstream infections (CLABSIs) in implanted catheters was elevated in 3 of the clinics compared to the other clinics.

CLABSIs frequently occur in pediatric hematology–oncology patients.^6,7^ Previous reports indicate that ambulatory pediatric oncology patients may experience a preventable hospitalization secondary to a CLABSI. Moreover, some children may require central line catheters removal because of CLABSIs that delay oncolytic therapy.^8,9^ For children with compromised immune systems, CLABSIs represent a life-threatening event.^9^

The goal of the second project was to reduce the incidence of ambulatory CLABSIs in children with implanted catheters. We also determined compliance with central line care best practice and CLABSI incidence at each St. Jude affiliate clinic before and after implementation of the interventions. Seven sites participated in this project.

### Methods

#### Program-wide Intervention.

In June 2015, 3 of the affiliate clinics had audit findings that revealed an unexpectedly high frequency of CLABSIs in ambulatory pediatric hematology and oncology patients. A core group of clinicians from these 3 sites reviewed relevant reports and established an ambulatory central line care bundle. They presented the bundle to the entire affiliate network for review. As with the time to antibiotics project, the information was provided to all sites via quarterly webinars and shared slide decks, and education was reinforced during monthly teleconferences.

The intervention bundle consisted of a checklist of structured processes (Table 1), designed by reports of best practices and deficiencies noted during clinical observations of central line care in the affiliate clinics.^10–13^ Some components of the bundle were already being used at many of the sites; however, a component of the bundle which was new at every site was the education of parents on central line care of implanted catheters (step 8 in Table 1). We added this education after we recognized that the implanted catheters were sometimes accessed in areas outside of the affiliate clinics, such as emergency departments, home care services, or radiology departments.

**Table 1.**
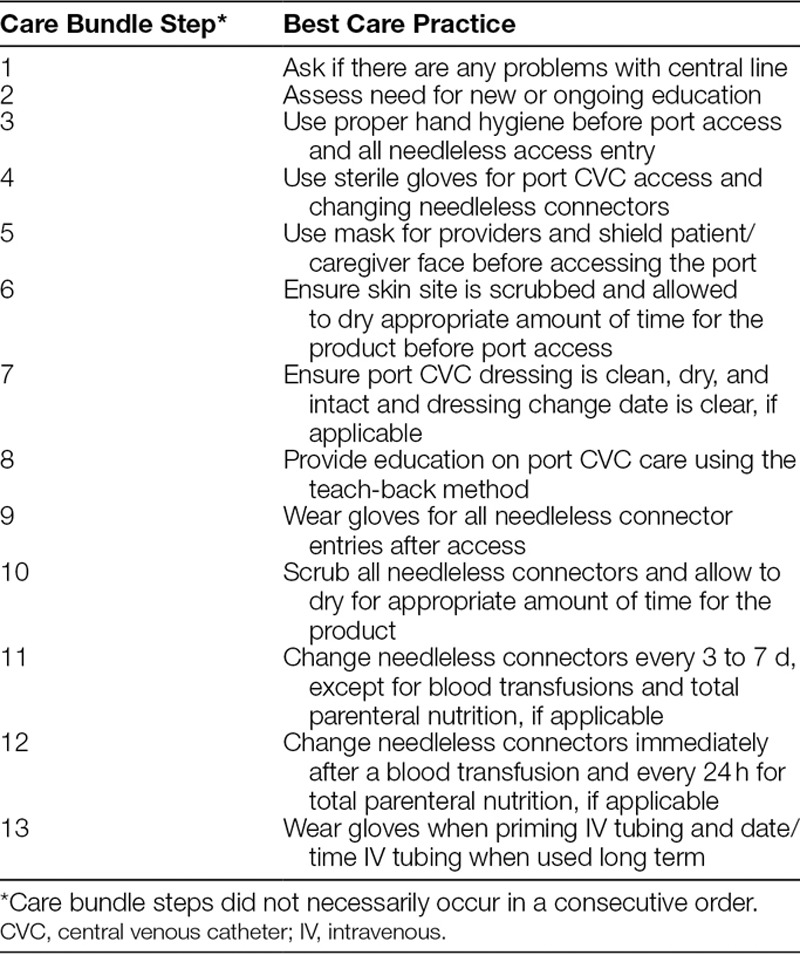
Ambulatory Port CVC Maintenance Care Bundle

### Site-specific Interventions

For children in the ambulatory setting with implanted catheters, clinic nurses implemented the care bundle at each site, and the medical director and nurse educator reviewed the importance of the care bundle during weekly team meetings. To assess compliance, a clinic nurse served as an observer for every port access event in the first 6 months of the intervention. This observer recorded whether or not each step in the care bundle was carried out. The affiliate nursing director captured all the results via a survey.

#### Study of Interventions

During the preintervention period for this project, June 2015 to February 2016, we reviewed the actual central line care practice occurring at each clinic site. This period also included a literature review and review of best practices of care for implanted catheters. All the affiliate clinics implemented the care bundle intervention between April and May 2017. The delay from February 2016 to April 2017 was due to the need to revise local institutional policies. We monitored the impact of our interventions from May 1, 2017 to November 30, 2017.

#### Measures

We measured compliance as the proportion of port access events in which clinic staff completed all components of the care bundle. We also collected data on specific steps for training purposes. Ambulatory CLABSI incidence was measured by extracting data from monthly nurse educator reports at each site. CLABSIs were defined according to the Center for Disease Control and National Healthcare Safety Network definitions. Incidence data reported here are from January 1, 2016 to May 31, 2018.

### Results

**Fig. 2.**
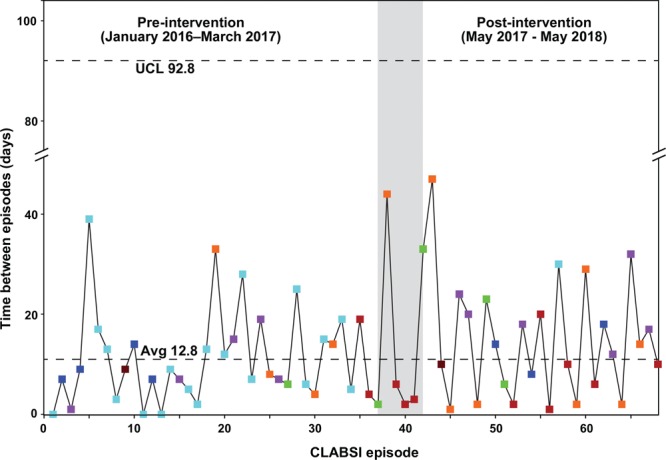
*Time* between successive CLABSI episodes before and after implementation of a line care bundle intervention. Data *is presented* as a statistical process control run chart for 68 successive CLABSI episodes across the network of affiliate sites. The gray column depicts the interval (April–May 2017) during which the different sites implemented the intervention. Black-dotted lines show the upper control limit and the mean value. Different colored symbols represent episodes at the different sites.

The incidence of CLABSIs in the preintervention and postintervention periods (pre/post) among the 7 sites that participated in this project were 2 (1/1), 5 (2/3), 7 (4/3), 11 (5/6), 11 (5/6), 12 (3/9), and 20 (19/1). The time between successive CLABSIs across all 7 sites is presented as a run chart^14^ in Figure 2, with the different sites represented by different-colored symbols. The mean time between CLABSIs was 12.8 days and did not show any changes when considered in the aggregate. In monthly assessments (a total of 361 observations), the proportion of port access events that were compliant with the intervention increased during the postintervention period, from 75% in June 2017, to 90%–100% in July through December 2017.

### Discussion

Our quality improvement intervention for reducing the incidence of CLABSIs was associated with apparent improvement in one of the sites (with 19 and 1 incidents in the preintervention and postintervention, respectively). In the remaining 6 sites, the small number of incidents, together with the inability to assess the CLABSI rate by line day data (which was not available) limited our ability to assess intervention efficacy. One challenge we encountered with the CLASBI project was the need to ensure the intervention complied with local institutional policies. This requirement led to a delay between the preintervention and postintervention periods. We addressed this challenge by emphasizing the importance of all institutions to meet national standards.^1,15,16^

## Conclusions

Most clinical quality improvement initiatives consist of a single-institution project, structured patient safety organizations, or large clinical networks governed by a similar infrastructure.^17–19^ For example, in the Children’s Hospital Association Hematology/Oncology CLABSI Collaborative, implementation of a standardized care bundle led to a ~30% reduction in mean CLABSI rate.^6^ However, quality improvement initiatives in the St. Jude affiliate network are not defined by any of these scenarios, but rather by an intermediate-size clinical network with separate institutional policies that work together to address joint problems in a similar patient population. Different remote locations and the absence of an integrated medical record system present challenges. Nonetheless, our results highlight how systematic quality improvement initiatives across a network of diverse sites can lead to improvements in patient care, and have potential implications for clinical networks in analogous situations.

Important take-home lessons from our experience are the value of education, the need to consider the diversity and context of the different sites, the ability of the different sites to learn from one another, the value of performance appraisal, and the opportunity for all stakeholders to participate. Education of the emergency department and after-hours staff had a significant impact on the time to antibiotic administration. Furthermore, information sharing among site-specific nurse educators was an essential component of recognizing that the process maps varied among sites, and therefore implementing site-specific interventions. Our platform for information sharing includes monthly teleconferences with nurse educators and St. Jude staff, and noncompulsory quarterly webinars with affiliate clinic physicians, nurses, pharmacists, and advanced care providers. Also, we share data electronically across sites, and we encouraged the clinic staff to review their data weekly. Finally, staff across the entire network are encouraged to participate in annual conferences—one for physicians and one for other healthcare professionals. These annual conferences emphasize train-the-trainer approaches, and although attendance is not compulsory, representatives from each affiliate site are always present.

The affiliate medical directors also found it useful to participate in performance appraisals, since the information gleaned from annual clinical audits guided site-specific internal training and processes. Affiliate medical directors also have the opportunity to initiate and lead quality improvement efforts, providing additional motivation that resonates across the entire network.

About stakeholder participation, our quality improvement experience included doctors, nurses, pharmacists, and parents, and allowed each of these groups to experience the opportunity to improve care. For example, the teach-back method provided to parents may help quality care throughout the patient experience including other departments outside of the affiliated clinic.^20,21^

Finally, we want to emphasize the importance of communication and transparency in quality improvement. Sharing of best practices, sharing of results, and sharing of improvements has brought together and reinforced our affiliate network. Similar approaches are likely to be helpful for many types of pediatric chronic illnesses.

## ACKNOWLEDGMENTS

The authors thank Nisha Badders, PhD, ELS, for scientific editing of the manuscript and Rebecca Quillivan for statistical support. The authors also thank affiliate site staff for their participation in the project and continuing drive for improving patient care.

## DISCLOSURE

The authors have no financial interest to declare in relation to the content of this article.

## Supplementary Material
