# Supplementary material for: Quality Improvement Interventions across a Network of Pediatric Hematology–Oncology Clinics
Source: Pediatr Qual Saf. 2019 Mar 11;4(2):e149. doi: 10.1097/pq9.0000000000000149 (PMC6494226; doi:10.1097/pq9.0000000000000149)
Supplement: Supplementary file 1 [file pqs-4-e149-s001.docx]

**SUPPLEMENTARY TABLE S1** Clinical observations during annual audits of affiliate clinics

| **Clinical documentation and observation metric** | **Source** |
| --- | --- |
| Staff read back verbal test results | JC; PC |
| Patient education before discharge with teach-back method | JC; ONS |
| Medical record documentation per institutional policy | JC; RC |
| Documentation of communication needs | JC; RC; EP |
| Documentation of age-appropriate pain assessment and reassessment | JC; RC; EP |
| Documentation of psychosocial assessment and reassessment each visit (≤ every 6 months); referrals made as necessary | APHON |
| Patient documentation includes functional assessment and reassessment each visit (≤ every 6 months) (JC policy: PC.01.02.01); referrals made as necessary | JC; PC |
| Patient documentation includes an abuse assessment each visit and follow-up if necessary (≤ every 6 months) | JC; PC |
| Patient documentation includes a physical assessment and reassessment each visit; describe assessment and appropriate interventions for real or potential alterations in protective mechanisms, mobility, elimination, ventilation, circulation, or comfort | APHON |
| Patient documentation of physical and mental health uses National Cancer Institute Common Terminology Criteria for Adverse Events | ONS |
| Orders for diagnostic and therapeutic tests and procedures, as well as their results, are documented | JC; RC |
| All diagnoses for conditions established during patient's course of care, treatment, services are documented | JC; RC |
| All medications ordered or prescribed during each visit are documented on the patient chart | JC; RC; EP |
| All chemotherapy administered has an "okay to give" order documented on the patient chart | ONS |
| Patient roadmaps are documented “up to date” on chart | ONS |
| All medication administration must include documentation of drug, dose, route, and response; chemotherapy includes documentation of blood return before and after administration; all medications require documentation of rate of administration; chemotherapy specifically requires start and stop times, regardless of type of administration (IVP or IVPB), and signature of two nurses who verify the chemotherapy | ONS |
| Documentation of the Hematology/Oncology Educational Needs Assessment results completed by both clinic and hospital staff; evidence of life-long learning by participating in ongoing educational activities (formal and independent) to expand knowledge, enhance role performance, and increase knowledge of professional issues | APHON |
| Documentation of flu vaccine administration to patients who are at least 6 months old; second dose should be administered at least 4 weeks later to patients who are immunocompromised | CDC |
| At registration, patients receive an armband, and two identifiers are used to place armbands; all staff perform positive patient identification before procedures and medication administration | JC; NPSG |
| Patient chart and computer information kept protected in all areas of clinic | JC |
| All staff washed hands or used alcohol hand cleaner before and after patient care | OSHA |
| Medications prepared by pharmacy and nursing staff are labeled correctly | JC; MM |
| Before all chemotherapy administration: (1) appropriate patient assessment is completed; (2) two nurses independently calculate patient specific drug dosages; verify written order for dosage, route, and mode of administration against protocol and roadmap; look for signed consent, “ok to give”, laboratory values and tests according to protocol; and (3) education of treatment with patient/caregiver is performed and verified | ONS |
| All staff use personal protective equipment appropriately when handling cytotoxic therapy | ONS; OSHA |
| Performs positive patient identification with two nurses at patient side for chemotherapy administration | JC; NPSG |
| Blood product administration is completed according to national standards | APHON |
| Patient/family education provides health teaching and anticipatory guidance about the disease and management in both the healthcare and home setting; teaching methods are appropriate to the situation, developmental level, learning needs, language preference, and culture; feedback is encouraged and teaching strategies are evaluated | APHON |
| Clinic monitors bacterial and fungal infections in their patient population | JC; IC |
| Evidence of monitoring central line–associated blood stream infections and interventions to reduce the number of incidences | CDC |
| Evidence of monitoring febrile neutropenia and a plan to assure antibiotic administration | IDSA |
| Evidence of two quality improvement projects per year | St. Jude |

APHON, Association of Pediatric Hematology Oncology Nursing; CDC, Centers for Disease Control and Prevention; EP, Element of Performance; IC, Infection Prevention and Control; IDSA, Infection Diseases Society of America; JC, Joint Commission; MM, Medication Management; NPSG, National Patient Safety Goals; ONS, Oncology Nursing Society; OSHA, Occupational Safety and Health Administration; PC, Provision of Care, Treatment, and Services; RC, Record of Care, Treatment and Services; St. Jude, St. Jude Children’s Research Hospital
